# Shiga Toxin-Producing *Escherichia coli* in Animal Reservoirs, Serotype Diversity, Virulence Profiles, and Antimicrobial Resistance Trends (2020–2026): A Scoping Review

**DOI:** 10.3390/antibiotics15090891

**Published:** 2026-09-11

**Authors:** Katarzyna Kosznik-Kwaśnicka, Agnieszka Necel, Tomasz Jarzembowski, Lidia Piechowicz

**Affiliations:** Department of Medical Microbiology, Faculty of Medicine, Medical University of Gdańsk, 80-204 Gdansk, Poland; katarzyna.kwasnicka@gumed.edu.pl (K.K.-K.); agnieszka.necel@gumed.edu.pl (A.N.); tjarzembowski@gumed.edu.pl (T.J.)

**Keywords:** STEC, EHEC, antibiotic resistance, Shiga toxin

## Abstract

**Background/Objectives**: Shiga toxin-producing *Escherichia coli* (STEC) are important zoonotic pathogens associated with gastrointestinal infections and hemolytic–uremic syndrome (HUS). Increasing serotype diversity, emergence of hybrid strains, and growing antimicrobial resistance complicate surveillance and treatment. This review summarizes recent data on STEC isolated from major animal reservoirs between 2020 and 2026. **Methods**: We comparatively analyzed peer-reviewed, open-access studies investigating STEC from cattle, sheep, goats, poultry, wildlife, and related animal products. We extracted and evaluated data on serotypes, virulence genes, hybrid pathotypes, and antimicrobial resistance determinants. **Results**: High serotype diversity was observed across all reservoirs, with non-O157 STEC frequently predominating over O157:H7. Ruminant isolates commonly carried *stx2* and additional virulence determinants associated with severe human disease, confirming cattle and small ruminants as major reservoirs of highly pathogenic STEC. Poultry-associated isolates showed greater variability in virulence profiles but frequently exhibited high rates of multidrug resistance. Hybrid strains combining virulence traits characteristic of multiple *E. coli* pathotypes were increasingly reported. Livestock-associated STEC commonly carried resistance determinants against β-lactams, tetracyclines, sulfonamides, quinolones, and polymyxins, whereas wildlife isolates generally showed lower rates of resistance. **Conclusions**: Animal-associated STEC populations demonstrate increasing genomic diversity, pathogenic potential, and antimicrobial resistance. The growing prevalence of non-O157 and hybrid strains highlights the limitations of traditional serotype-focused surveillance and emphasizes the need for integrated One Health monitoring strategies.

## 1. Introduction

Shiga toxin-producing *Escherichia coli* (STEC) are among the most important zoonotic foodborne pathogens worldwide and cause a wide spectrum of human diseases, ranging from mild diarrhea to hemorrhagic colitis and hemolytic uremic syndrome (HUS) [1,2]. According to recent reports from the European Food Safety Authority (EFSA) and the European Center for Disease Prevention and Control (ECDC), STEC infections remain a significant public health concern, with thousands of confirmed cases reported annually and hospitalization rates exceeding those observed for several other major bacterial enteric pathogens [3]. The clinical severity of STEC infections is primarily associated with the production of Shiga toxins (*Stx*), potent cytotoxins capable of causing systemic vascular damage and life-threatening complications.

Historically, surveillance and outbreak investigations have focused predominantly on serotype O157 due to its frequent association with severe disease and large foodborne outbreaks [4,5]. However, increasing evidence indicates that numerous non-O157 STEC serotypes contribute substantially to human infections worldwide [6]. Advances in molecular typing and whole-genome sequencing have revealed extensive genetic diversity within STEC populations and facilitated the identification of recently recognized hybrid strains that combine virulence characteristics from multiple *E. coli* pathotypes [7,8]. These findings challenge traditional serotype-based surveillance approaches and highlight the need to more broadly characterize STEC populations circulating in animal and environmental reservoirs.

**Figure 1:**
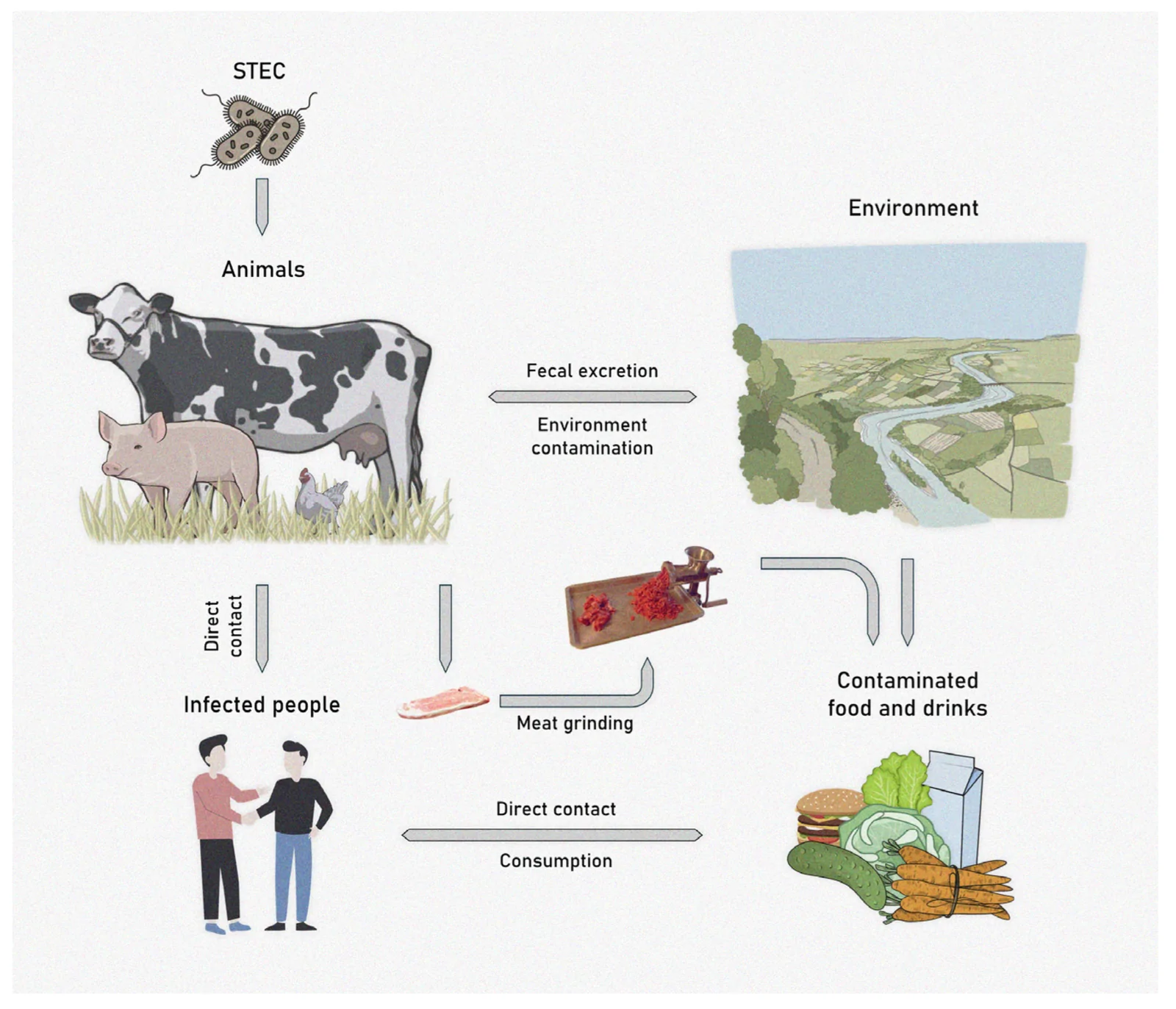
Transmission pathways and reservoirs of Shiga toxin-producing *Escherichia coli* (STEC). Created in MS PowerPoint 365 by the authors.

Animals represent the principal reservoir of STEC and play a central role in the maintenance and transmission of these pathogens. Ruminants, particularly cattle, are recognized as the primary asymptomatic carriers, although STEC has also been reported in sheep, goats, poultry, wildlife, and game animals [5,9]. STEC shedding through animal feces contributes to contamination of food products, water sources, and agricultural environments, creating multiple pathways for human exposure (Figure 1). Consequently, understanding the diversity and distribution of STEC within animal reservoirs is essential for risk assessment and the development of effective prevention strategies.

In addition to concerns about virulence, increasing attention has focused on the antimicrobial resistance (AMR) profiles of STEC isolates. Although antimicrobial therapy is generally not recommended for STEC infections because certain antibiotics may enhance Shiga toxin production [10], the presence of resistance determinants remains epidemiologically important [11]. Animal-associated STEC populations may serve as reservoirs of transferable resistance genes, contributing to the broader dissemination of AMR within microbial communities. Recent studies have reported resistance to β-lactams, tetracyclines, sulfonamides, quinolones, and even critically important antimicrobials such as polymyxins, emphasizing the need to integrate virulence and resistance surveillance within a One Health framework [12].

This review summarizes recent knowledge on STEC isolated from major animal reservoirs between 2020 and 2026, with particular emphasis on serotype diversity, virulence characteristics, recently recognized hybrid pathotypes, and antimicrobial resistance profiles. By comparing findings across livestock, poultry, and wildlife reservoirs, this review provides an updated overview of the epidemiology of animal-associated STEC and highlights challenges relevant to surveillance, food safety, and public health.

## 2. Materials and Methods

A structured literature search was performed to identify recent publications on STEC associated with major animal reservoirs, focusing on serotype diversity, virulence characteristics, hybrid pathotypes, antimicrobial resistance, and genomic features. The aim was to provide a narrative synthesis rather than a formal systematic review or meta-analysis.

A literature search was conducted using the PubMed and Web of Science databases to identify studies published between January 2020 and March 2026 concerning Shiga toxin-producing *Escherichia coli* (STEC) isolated from animal reservoirs. Search terms included combinations of keywords related to STEC and animal hosts: (“Shiga toxin-producing *Escherichia coli*” OR STEC OR EHEC) AND (cattle OR bovine OR sheep OR goat OR poultry OR wildlife OR game animals) AND (virulence OR antimicrobial resistance OR serotype OR genomics).

Open-access availability was retained as an eligibility criterion. This criterion was deliberately applied to ensure that the publications contributing to the review could be accessed in full text and independently examined by readers without subscription or institutional access. The authors acknowledge that restricting eligibility to openly accessible publications may exclude relevant subscription-based studies and may therefore introduce selection bias.

**Figure 2:**
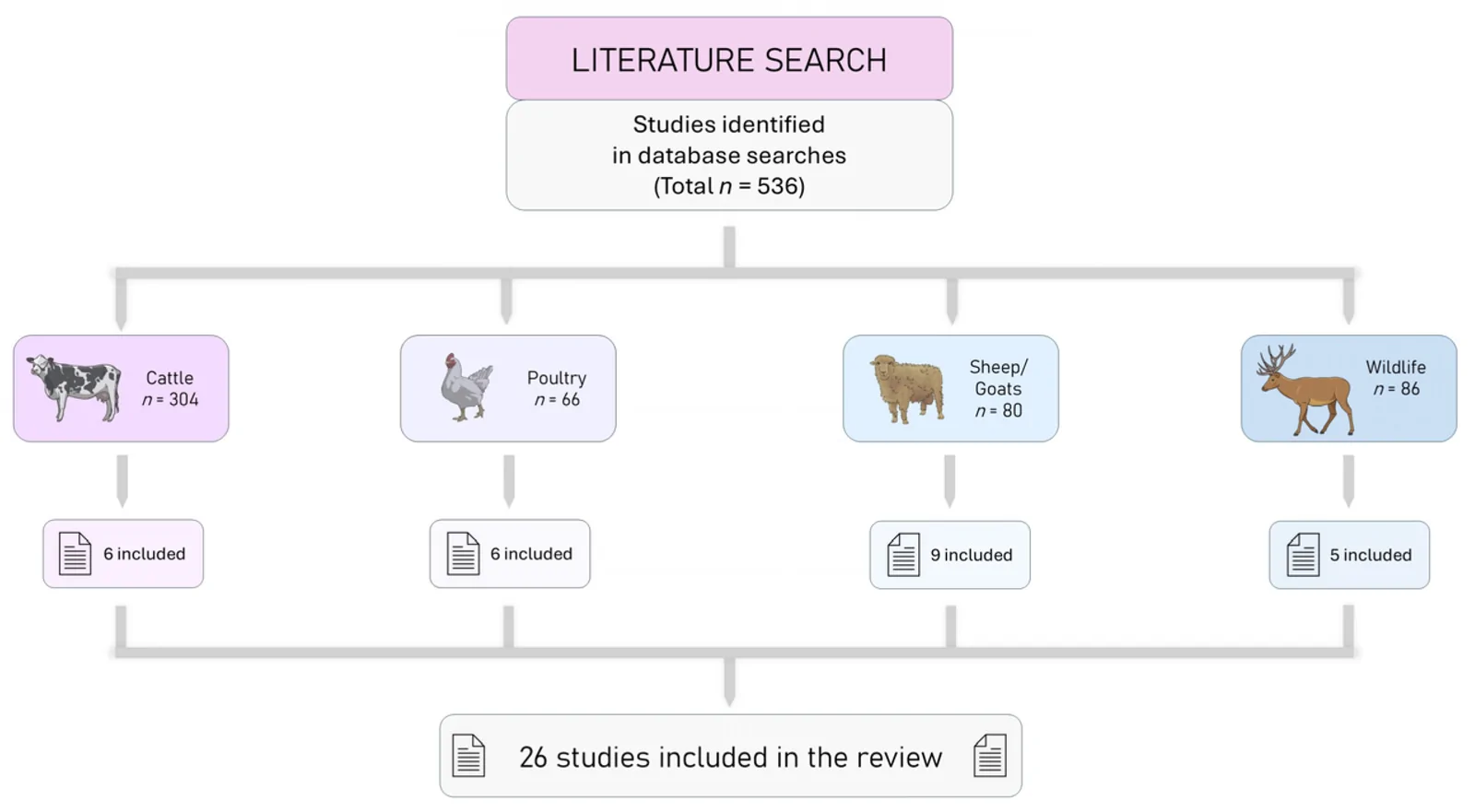
The overview of the literature search and study selection.

Records were managed in Rayyan, with duplicates removed and publications assessed for relevance and eligibility. Eligible studies were original, peer-reviewed, open-access research investigating confirmed STEC associated with animals, animal-derived products, secretions, or animal-associated environments. 26 studies were included in the final narrative synthesis (Figure 2). Due to substantial methodological heterogeneity among the included studies, including differences in geographical setting, sampling strategies, sample sizes, detection methods, virulence-gene panels, and antimicrobial susceptibility-testing procedures, findings were synthesized narratively rather than through formal meta-analysis. Graphical summaries were intended to illustrate patterns reported across individual studies rather than pooled estimates.

## 3. Comparative Analysis of STEC Reservoirs

### 3.1. Serotype Diversity Across Animal Reservoirs

Substantial serotype diversity was observed across all animal reservoirs included in this review, although the composition and dominant lineages varied considerably by host species and geographic region (Table 1). Although STEC surveillance has historically focused primarily on O157, recent studies consistently demonstrate that non-O157 serotypes constitute the majority of circulating strains in livestock, poultry, and wildlife populations [13,14].

Cattle exhibited the highest overall serotype diversity and remain the principal reservoir of STEC. Individual studies identified 53 to 72 serotypes within single cattle populations, highlighting substantial heterogeneity among bovine-associated STEC [15,16,17,18,19,20]. Although O157 was detected in most regions, it was rarely the dominant serotype. Instead, geographically distinct serotypes predominated, including O82 in South Africa [20], O29 in Portugal [15], and O22 and O8 in Pakistan [19]. Several members of the clinically important “Big Seven” STEC group, including O26, O103, O111, and O157, were also frequently identified, confirming the importance of cattle as reservoirs of strains with recognized zoonotic potential [15,16,17,20].

Similarly, sheep and goats harbored highly diverse STEC populations dominated by non-O157 serotypes. The most frequently reported serotypes were O91, O128, O146, O103, and O76, though substantial regional variation was observed. Studies from Ireland and the Netherlands identified O91, O128, and O146 as dominant serotypes [21,22], whereas South African goat populations were characterized by O103 and O3 [23,24]. Notably, O157 was often detected only sporadically or was absent, further emphasizing the epidemiological importance of non-O157 STEC within small-ruminant reservoirs [8,21,22,25,26].

In contrast, poultry-associated STEC populations showed lower overall diversity and stronger regional specificity. Serotypes such as O78, O15, O2, and O91 predominated in Egyptian poultry production systems [27], while O177 was identified as a characteristic lineage among South African broiler isolates [28]. Although O157 was reported in poultry from Nigeria and Iran, these findings were generally less frequent than those reported in cattle and small-ruminant studies [23,29,30]. The observed serotype composition suggests that poultry may contribute to the dissemination of specific regional STEC lineages rather than serving as a major reservoir of globally distributed serotypes.

Wildlife-associated STEC generally occurred at lower prevalence than in livestock reservoirs but demonstrated considerable serotype diversity. European game animals and game meat frequently carried serotypes such as O146, O110, O27, and O21 [31,32], whereas Japanese wildlife populations were characterized by genotypes including O146, O11, and O54 [33,34]. Several wildlife-associated serotypes were rarely reported in domestic livestock, suggesting the existence of partially distinct ecological populations of STEC maintained within natural ecosystems [31,32,33,34,35]. These findings indicate that wildlife may contribute to the environmental persistence and dissemination of genetically diverse STEC lineages.

**Figure 3:**
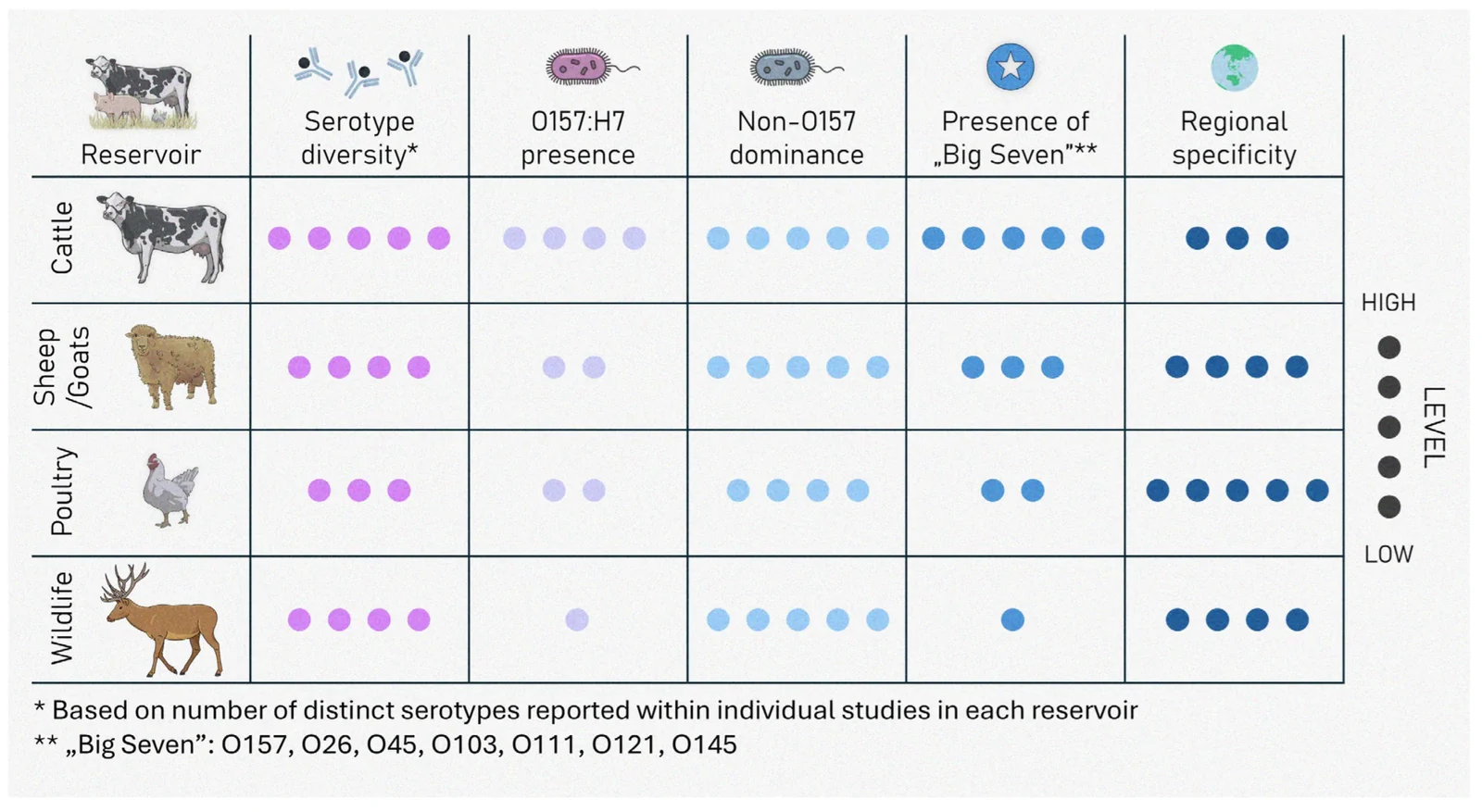
Serotype diversity across reservoirs (2020–2026). Created in MS PowerPoint 365 by the authors.

Overall, the reviewed studies demonstrate that animal-associated STEC populations exhibit extensive serotype heterogeneity, with non-O157 strains predominating across most reservoirs (Figure 3). The marked regional variation observed across host species highlights the limitations of surveillance strategies focused solely on a restricted number of serogroups and supports the implementation of broader molecular approaches for STEC monitoring.

### 3.2. Virulence Profiles

The distribution of Shiga toxin genes (*stx*) varied substantially among animal reservoirs. Cattle-associated STEC were characterized by a predominance of *stx2*-positive isolates [15,20]. Simultaneous carriage of *stx1* and *stx2* occurred most commonly, suggesting high pathogenic potential in bovine-associated STEC populations [24,25,36]. In contrast, sheep and goat isolates frequently exhibited a more balanced distribution of toxin genes, with some studies reporting predominance of *stx1* [8,24,26], while others identified *stx2* as the dominant subtype [25,36]. Poultry-associated STEC displayed marked geographical variability [27,29,36], whereas wildlife isolates were predominantly single-*stx2* subtypes and comparatively rare in *stx1*/*stx2* combinations [8,31,33,34].

Successful colonization of the host intestinal tract depends on multiple adhesion-associated determinants. The intimin-encoding gene *eae*, considered the most important in attaching-and-effacing lesions, was detected mostly in cattle-associated isolates [8,15,20,34], while it was relatively uncommon among sheep, goat, poultry, and wildlife isolates [8,21,22,27,29,36]. The low prevalence of *eae* in many reservoirs suggests that alternative adherence mechanisms may also play a role in STEC ecology.

Among these alternative factors, *lpfA* (Long Polar Fimbriae) and *iha* (IrgA-homolog adhesin) were frequently detected in isolates from both livestock and wildlife, particularly among *eae*-negative strains [19,22,31]. These findings support previous observations that STEC populations circulating in healthy animal reservoirs often rely on adhesion mechanisms distinct from the classical EHEC paradigm.

The enterotoxigenic hemolysin (*ehxA*) gene was among the most consistently detected accessory virulence factors across all reservoirs. High frequencies were reported in cattle, sheep, and goat isolates, often exceeding 70% among characterized strains [8,15,19,20,21,22,24,27,33,34,36]. The widespread distribution of *ehxA* among ruminant-associated STEC suggests that this determinant is a stable component of their virulence.

Similarly, the increased serum survival gene *iss* was detected in multiple reservoirs, including livestock and wildlife isolates [8,19,22,27,31,33,34]. Its frequent occurrence suggests a potential role in bacterial persistence and adaptation within animal hosts, although its contribution to human disease remains less clearly defined than that of Shiga toxins.

**Figure 4:**
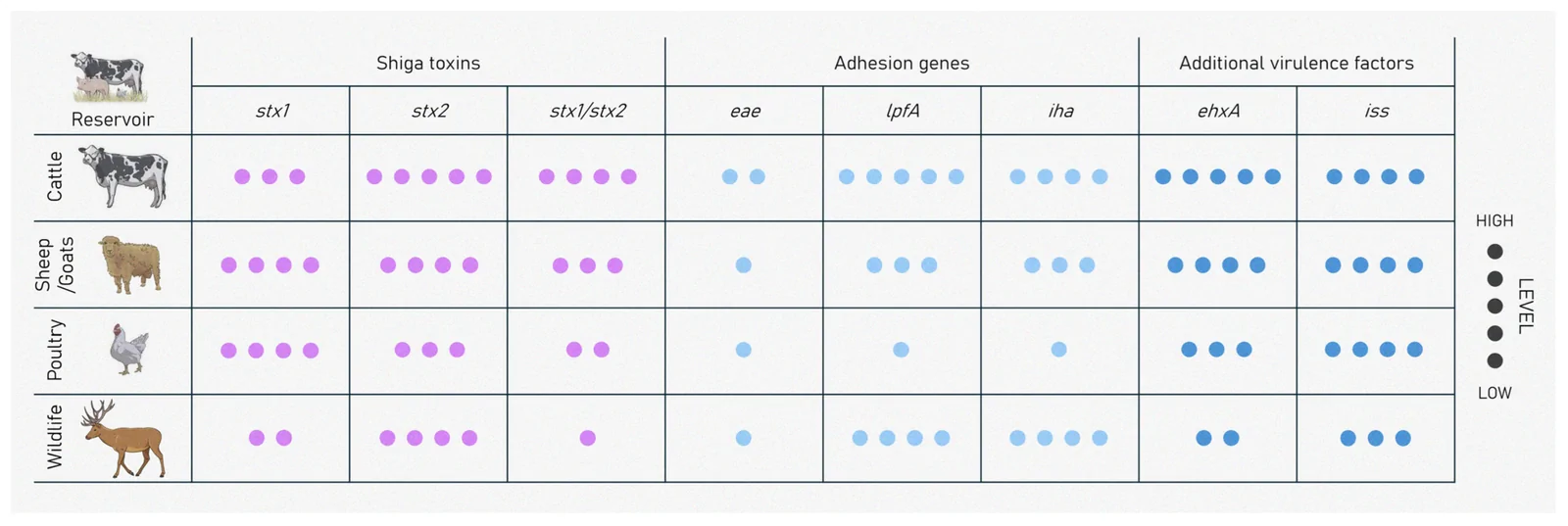
Comparative virulence profiles of STEC across animal reservoirs (2020–2026). Created in MS PowerPoint 365 by the authors.

Collectively, cattle-associated STEC exhibited the highest virulence potential, characterized by frequent *stx2* carriage, common *stx1*/*stx2* combinations, and high prevalence of accessory virulence determinants such as *ehxA* and *iss*. Sheep and goats displayed comparable virulence gene repertoires, although the lower prevalence of *eae* suggests a greater reliance on alternative adherence mechanisms. Poultry isolates showed considerable heterogeneity, whereas wildlife-associated STEC were generally characterized by simpler virulence profiles dominated by single toxin subtypes and fewer classical adhesion determinants (Figure 4).

These findings indicate that virulence potential varies substantially among reservoirs and cannot be predicted accurately based solely on serotype. Consequently, surveillance strategies should increasingly incorporate virulence profiling alongside traditional serotyping approaches.

### 3.3. Antimicrobial Resistance

Phenotypic antimicrobial resistance varied considerably among animal reservoirs but consistently showed that livestock-associated STEC populations carry a substantially greater burden of resistance than wildlife isolates (Figure 4). Resistance to β-lactam antibiotics represented the most frequently reported phenotype across the reviewed studies. Poultry and small-ruminant isolates showed particularly high rates [29,30,36,37,38], with several studies reporting complete resistance to ampicillin or amoxicillin-containing formulations [36,38]. Cattle-derived STEC also exhibited high β-lactam resistance, although prevalence varied geographically [17,18,27]. In contrast, wildlife-associated isolates from Europe and Japan generally remained susceptible to β-lactam antibiotics [31,34].

Resistance to tetracyclines was also widespread, particularly among poultry and sheep/goat isolates, with rates frequently exceeding 40–50% of tested isolates [17,23,26,36]. Similar trends were observed for sulfonamides, especially in small-ruminant populations, whereas cattle generally exhibited lower frequencies [18,26,37]. Quinolone resistance showed greater geographical variability, with high nalidixic acid resistance reported in South African and Iranian livestock [23,29,36], while resistance to ciprofloxacin remained comparatively uncommon in most studies, with the highest number of resistant isolates (25.8%) reported from poultry samples from Nigeria [29,30,36].

**Figure 5:**
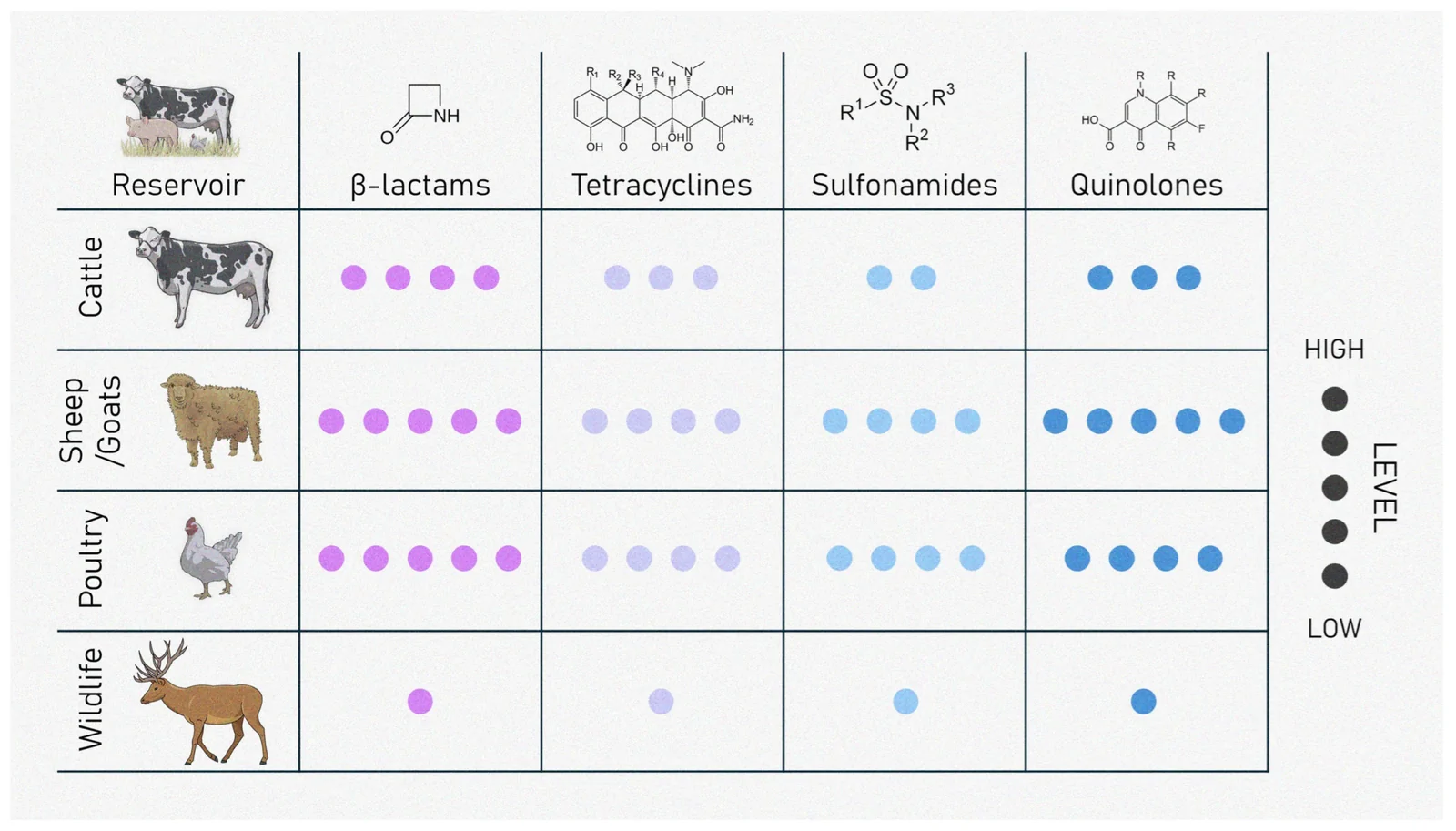
Comparative phenotypic antimicrobial resistance of STEC by reservoir (2020–2026). Created in MS PowerPoint 365 by the authors.

Overall, the reviewed data indicate that intensive livestock production systems, particularly poultry and small-ruminant production, represent the reservoirs with the greatest phenotypic antimicrobial resistance burden, whereas wildlife continues to serve as a relatively antimicrobial-susceptible reservoir (Figure 5).

The observed phenotypic resistance patterns were largely supported by the distribution of acquired antimicrobial resistance genes. Among β-lactam resistance determinants, *blaTEM* and *blaCTX-M* variants were the most frequently reported, particularly among cattle, poultry, and sheep-derived isolates [23,26,27,36,37], indicating the continued dissemination of clinically relevant β-lactam resistance mechanisms within animal reservoirs.

Genes conferring resistance to tetracyclines (*tetA*) and sulfonamides (*sul1* and *sul2*) were among the most consistently detected resistance determinants. Their high prevalence closely mirrored the widespread phenotypic resistance observed in these antimicrobial classes, particularly among poultry and small-ruminant STEC isolates [17,23,26,27,36].

One of the most concerning findings was the detection of plasmid-mediated colistin resistance genes in communal goat populations from South Africa. Both *mcr-1* and *mcr-4* were identified at high frequencies, exceeding those reported in comparable livestock populations from other regions [26,36]. Considering that colistin remains a critically important antimicrobial for human medicine, these findings warrant continued surveillance of mobile resistance determinants in livestock-associated STEC.

Multidrug resistance (MDR) was common among livestock-associated STEC but varied substantially between reservoirs and geographical regions. The highest MDR prevalence was reported among South African goat isolates (94.1%), followed by cattle isolates from Egypt (89%) and poultry-derived isolates from South Africa and Nigeria, with MDR exceeding 70% in several studies [17,19,27,30,36]. In contrast, wildlife-associated STEC generally exhibited low resistance burdens and rarely carried acquired resistance determinants beyond intrinsic chromosomal resistance genes, like the *blaEC* gene, corresponding to class C cephalosporinases, and the AcrAB–TolC multidrug efflux system [31,33,34].

Collectively, the reviewed evidence indicates that antimicrobial resistance in STEC is primarily associated with livestock production rather than wildlife reservoirs. While cattle remain the principal reservoir of STEC overall, poultry and small ruminants frequently harbor isolates exhibiting the highest resistance levels and MDR prevalence. The emergence of ESBL-associated genes and plasmid-mediated colistin resistance further highlights the importance of integrated surveillance programs combining antimicrobial stewardship with molecular monitoring of resistance determinants in animal populations.

### 3.4. Hybrid Strains Recognition

**Figure 6:**
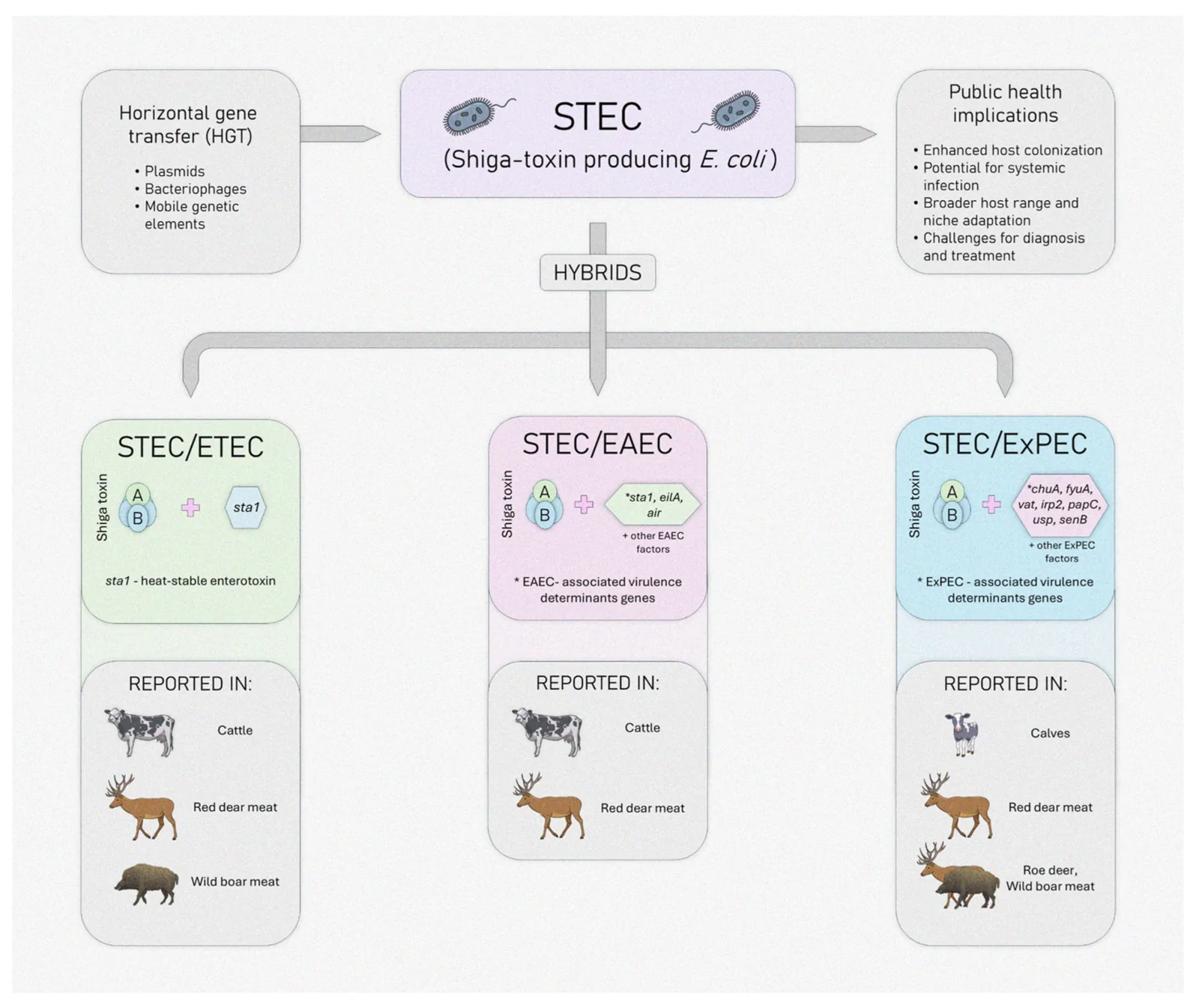
Emergence of hybrid STEC pathotypes through acquisition of virulence determinants from other pathogenic *E. coli* lineages. Created in MS PowerPoint 365 by the authors.

The genomic plasticity of *E. coli* enables the emergence of hybrid pathotypes that combine virulence determinants typically associated with distinct diarrheagenic or extraintestinal lineages [39]. Through horizontal gene transfer mediated by plasmids, bacteriophages, and pathogenicity islands, STEC strains may acquire additional virulence traits that could enhance host colonization, environmental persistence, or disease severity [40] (Figure 6).

STEC/ETEC hybrids represented the most widely distributed hybrid category identified in the reviewed literature. These strains combine Shiga toxin genes with enterotoxigenic determinants, particularly the heat-stable enterotoxin gene *sta1*. Such hybrids have been reported in wildlife-derived isolates from European red deer and wild boar populations [31].

STEC/EAEC hybrids, often referred to as enteroaggregative hemorrhagic *E. coli* (EAHEC), combine Shiga toxin production with virulence factors characteristic of enteroaggregative *E. coli*. An OX:H10 isolate recovered from cattle in Iran carried both *stx2* and EAEC-associated determinants [8], while O27:H30 isolates recovered from European game meat possessed additional EAEC-associated genes, including *pic*, *eilA*, and *air* [31]. Although these strains did not always harbor the complete EAEC virulence repertoire, their existence demonstrates ongoing genetic exchange between STEC and aggregative lineages.

Particularly concerning are STEC/ExPEC hybrids carrying virulence determinants associated with extraintestinal pathogenic *E. coli*. Several wildlife-derived isolates, including O110:H31 and O146:H28 recovered from European game meat, possessed ExPEC-associated genes such as *chuA*, *fyuA*, *vat*, *irp2*, and *papC* [16,31]. These factors are linked with iron acquisition, increased serum survival, and extraintestinal persistence, suggesting an enhanced capacity for systemic infection. Similar hybrid traits have been reported in the emerging O80:H2 lineage, which is associated with severe disease in both humans and calves [16]. The overview of reported hybrid STEC pathotypes is presented in Table 2.

The emergence of hybrid STEC pathotypes challenges traditional classifications based solely on serotype or Shiga toxin production. Several hybrid strains identified in livestock and wildlife reservoirs combine virulence determinants for intestinal colonization, toxin production, and extraintestinal survival. Consequently, risk assessment strategies that focus exclusively on classical STEC markers may underestimate the pathogenic potential of these emerging lineages. The increasing use of whole-genome sequencing is expected to reveal additional hybrid variants and improve understanding of their epidemiological significance.

## 4. Discussion

The studies published between 2020 and 2026 demonstrate that the epidemiology of Shiga toxin-producing *Escherichia coli* (STEC) in animal reservoirs is becoming increasingly complex and heterogeneous. Across cattle, small ruminants, poultry, and wildlife, the reviewed literature consistently revealed high serotype diversity, broad variability in virulence gene content, growing recognition of hybrid strains, and dissemination of antimicrobial resistance determinants. Importantly, these observations further support the gradual shift from the traditional O157:H7-centered perspective toward recognition of non-O157 and hybrid STEC lineages as major epidemiological and public health concerns [41,42,43].

Historically, STEC surveillance and diagnostics have focused primarily on O157:H7 because of its strong association with hemorrhagic colitis and hemolytic uremic syndrome (HUS) [44]. However, the reviewed studies clearly demonstrate that O157:H7 now frequently represents only a minority of isolates recovered from animals. In cattle, numerous non-O157 serotypes, including O22:H16, O91:H8, O113:H21, O181:H8, and O26:H11, were commonly detected, with similar trends observed in goats, sheep, poultry, and wildlife. Particularly noteworthy was the predominance of HUS-associated O113:H21 in backyard poultry from Chile and the recurrent identification of serotypes such as O146:H21, O128:H2, O91:H14, and O76:H19 in small ruminants and game meat [31,38].

These findings are consistent with previous epidemiological studies conducted before 2020, which suggested increasing global importance of non-O157 STEC strains in both human infections and animal reservoirs [6,45]. Earlier surveillance data from Europe and North America demonstrated that serogroups O26, O45, O103, O111, O121, and O145 were increasingly associated with severe human disease and outbreaks, leading to their classification among the “Big Six” non-O157 STEC serogroups [46]. The present review expands these observations by showing that current animal-associated STEC populations extend far beyond traditionally monitored serogroups and include numerous rare, emerging, and hybrid lineages that may remain undetected by serotype-focused diagnostic approaches.

An important finding reported across the reviewed studies is variation in virulence-associated characteristics between ruminant- and poultry-associated STEC isolates. In cattle, sheep, and goats, *stx2-*positive isolates frequently predominated and were often accompanied by additional virulence determinants, including *eae*, *ehxA*, *lpfA*, and *toxB*. Since *stx2*, particularly subtypes *stx2a* and *stx2c*, is strongly associated with severe human disease and HUS, these findings confirm ruminants as the major reservoir of highly virulent STEC strains with substantial zoonotic potential [47,48]. In contrast, poultry-derived STEC generally showed more heterogeneous virulence profiles and lower prevalence of high-risk virulence combinations. Although *stx1* and *stx2* were commonly detected, adhesion-associated genes such as *eae* were usually less frequent and varied considerably between regions, leading to the conclusion that poultry-associated STEC may have lower zoonotic potential than their ruminant-derived counterparts.

Further focus on the *eae* gene revealed substantial differences between the discussed animal reservoirs. In several cattle and wildlife studies, *eae* was absent or detected only rarely, whereas some Belgian and Iranian isolates demonstrated nearly universal carriage. Similar heterogeneity was observed among ovine, caprine, and poultry isolates [8,16]. Such findings align with previous literature indicating that STEC pathogenicity cannot be explained solely by the presence of *eae* [49]. Indeed, several severe outbreaks have been linked to *eae-*negative STEC strains, particularly hybrid pathotypes that carry alternative adhesion systems or additional toxins [50,51]. The widespread identification of accessory virulence determinants such as *ehxA*, *lpfA*, *tir*, *toxB*, *iss*, *iha*, *subA*, and numerous type III secretion-associated genes further supports the concept that STEC pathogenic potential results from highly variable combinations of virulence traits rather than single markers alone.

The occurrence of hybrid STEC strains represents an important feature reported in the reviewed studies. Hybrid strains carrying virulence determinants characteristic of other *E. coli* pathotypes, including enterotoxigenic (ETEC) and extraintestinal pathogenic (ExPEC) lineages, were detected in isolates from cattle, sheep, goats, and wildlife [19,22,31]. Similar hybridization events have previously attracted major attention following the 2011 German O104:H4 outbreak, during which an enteroaggregative *E. coli* strain acquired *stx-*encoding phages and caused one of the largest HUS outbreaks in Europe [39,51]. The increasing detection of comparable hybrid strains in animal reservoirs suggests ongoing genomic recombination and horizontal gene transfer within environmental *E. coli* populations. Consequently, reliance on classical serotyping may no longer provide sufficient information for accurate risk assessment, emphasizing the growing importance of whole-genome sequencing-based surveillance approaches [7].

Comparison of antimicrobial resistance patterns between animal groups revealed notable differences likely associated with varying levels of antibiotic exposure. Livestock-associated STEC, particularly those isolated from cattle, sheep, goats, and poultry, frequently exhibited high multidrug resistance (MDR) rates and carried resistance determinants against β-lactams, tetracyclines, sulfonamides, aminoglycosides, quinolones, and polymyxins. Resistance genes such as *blaTEM*, *blaCTX-M*, *tetA*, *sul1*/*sul2*, *aadA1*, and *mcr-1* were repeatedly identified across geographically distant regions [23,26,29,30]. Particularly concerning was the detection of extended-spectrum β-lactamase (ESBL)-associated genes and colistin resistance determinants in ovine and poultry isolates, since both antimicrobial classes are considered critically important for human medicine [52].

Recent studies of livestock-associated STEC have reported multidrug resistance (MDR), including resistance to multiple antimicrobial classes such as tetracyclines, sulfonamides, and β-lactams [53]. This trend likely reflects continued antimicrobial pressure in animal production systems and parallels global observations reported by WHO and EFSA [52,54]. Importantly, many detected resistance determinants, including ESBL- and colistin-associated genes, are plasmid-mediated and therefore can be horizontally transferred between bacterial populations.

In contrast, wildlife-derived STEC generally exhibited lower resistance levels and lacked the extensive MDR profiles commonly observed in livestock isolates. Resistance in wildlife was mainly associated with intrinsic mechanisms such as AcrAB–TolC and *mdfA* efflux systems, likely reflecting lower antibiotic exposure in natural environments [55]. Nevertheless, wildlife may still contribute to environmental dissemination of STEC and resistance-associated genes through ecological transmission networks.

Overall, cattle remain the principal reservoir of highly diverse STEC populations and likely represent the major source of foodborne transmission [56]. Small ruminants also constitute important reservoirs due to the frequent occurrence of *stx2*-positive and MDR isolates [57]. Poultry, although historically considered a secondary reservoir, demonstrated high resistance rates and substantial virulence diversity in several recent studies, suggesting that their epidemiological role may currently be underestimated and may require future monitoring.

Collectively, the reviewed studies highlight the relevance of integrated One Health surveillance incorporating animal, environmental, and human health perspectives. The diversity of STEC characteristics reported across the included studies indicates that reliance on O157:H7 identification or limited virulence screening alone may overlook some STEC variants. Incorporating serotype, virulence, antimicrobial resistance, and genomic information may provide additional information for risk assessment and outbreak investigation [7,31,47]. Furthermore, continued monitoring of antimicrobial resistance in STEC is particularly important given the ongoing debate surrounding antibiotic use during STEC infections and the risk of Shiga toxin induction associated with certain antimicrobial classes. Ultimately, the increasing genomic plasticity, ecological adaptability, and resistance burden of STEC emphasize the need for globally coordinated surveillance systems capable of rapidly detecting emerging pathogenic lineages before they become major public health threats.

Several limitations should be considered when interpreting the findings of this review. The included studies varied substantially in geographic location, animal populations, sample size, sampling strategies, STEC detection and characterization methods, virulence genes investigated, and antimicrobial susceptibility testing procedures. Consequently, apparent differences between animal reservoirs may partly reflect methodological differences rather than true differences in STEC characteristics. In addition, not all studies investigated the same virulence or antimicrobial-resistance determinants, and the absence of a reported determinant was therefore not interpreted as evidence of its absence. The open-access eligibility criterion may have further limited the available evidence and introduced selection bias. Finally, because the review was designed as a narrative synthesis, quantitative pooling and formal temporal or cross-reservoir comparisons were not performed.

## Figures and Tables

**Table 1:** Regional variation in predominant STEC serotypes.

Reservoir	Observed Variation	Most Commonly Occurring Serotypes
**Cattle**	Highest diversity	O82:H8, O113:H21, **O157:H7**, O29:H12, O111:H8
**Sheep/Goats**	Non-O157 dominant	O91:H14, O128:H2, O146:H21, O103:H8, O76:H19
**Poultry**	Region-specific	O78, O177, O2:H6, O91:H21, **O157:H7**
**Wildlife**	Low prevalence, broad diversity	O146:H28, O110:H31, O146, O11, O27:H30

**Table 2:** Representative hybrid STEC pathotypes identified in animal reservoirs (2020–2026).

Hybrid Type	Representative Serotype	Additional Virulence Traits	Reservoir	Region
STEC/ETEC	O187:H28	*sta1*	Red deer meat	Austria
STEC/EAEC	OX:H10	Enteroaggregative markers	Cattle	Iran
STEC/EAEC	O27:H30	*pic*, *eilA*, *air*	Red deer meat	Europe
STEC/ExPEC	O80:H2	*hlyF*	Calves	Europe
STEC/ExPEC	O110:H31	*chuA*, *fyuA*, *vat*, *irp2*, *papC*	Roe deer meat	Switzerland
STEC/ExPEC	O146:H28	*chuA*, *usp*, *senB*	Roe deer, Boar	Europe

Note: *sta1*—heat-stable enterotoxin 1, *pic*—serine protease, *eilA*—HilA-like regulator, *air*—enteroaggregative immunoglobulin repeat protein, *hlyF*—hemolysin F, *chuA*—outer membrane hemin receptor, *fyuA*—siderophore receptor, *vat*—vacuolating autotransporter, *irp2*—yersiniabactin, *papC*—P fimbriae, *usp*—uropathogenic-specific protein, *senB*—plasmid-encoded enterotoxin.

## Data Availability

No new data were created or analyzed in this study. Data sharing is not applicable to this article.
